# Applications of Bayesian shrinkage prior models in clinical research with categorical responses

**DOI:** 10.1186/s12874-022-01560-6

**Published:** 2022-04-28

**Authors:** Arinjita Bhattacharyya, Subhadip Pal, Riten Mitra, Shesh Rai

**Affiliations:** 1 Department of Bioinformatics & Biostatistics, University of Louisville, Louisville, KY USA; 2 Biostatistics & Bioinformatics Facility, JG Brown Cancer Center, University of Louisville, Louisville, KY USA; 3 The Christina Lee Brown Envirome Institute, University of Louisville, Louisville, KY USA; 4 University of Louisville Alcohol Research Center, University of Louisville, Louisville, KY USA; 5 University of Louisville Hepatobiology & Toxicology Center, University of Louisville, Louisville, KY USA

**Keywords:** Shrinkage priors, Logistic regression, Horseshoe, Dirichlet Laplace, MCMC, Polya-Gamma, Multinomial, ADNI, Pima, Data augmentation

## Abstract

**Background:**

Prediction and classification algorithms are commonly used in clinical research for identifying patients susceptible to clinical conditions such as diabetes, colon cancer, and Alzheimer’s disease. Developing accurate prediction and classification methods benefits personalized medicine. Building an excellent predictive model involves selecting the features that are most significantly associated with the outcome. These features can include several biological and demographic characteristics, such as genomic biomarkers and health history. Such variable selection becomes challenging when the number of potential predictors is large. Bayesian shrinkage models have emerged as popular and flexible methods of variable selection in regression settings. This work discusses variable selection with three shrinkage priors and illustrates its application to clinical data such as Pima Indians Diabetes, Colon cancer, ADNI, and OASIS Alzheimer’s real-world data.

**Methods:**

A unified Bayesian hierarchical framework that implements and compares shrinkage priors in binary and multinomial logistic regression models is presented. The key feature is the representation of the likelihood by a Polya-Gamma data augmentation, which admits a natural integration with a family of shrinkage priors, specifically focusing on Horseshoe, Dirichlet Laplace, and Double Pareto priors. Extensive simulation studies are conducted to assess the performances under different data dimensions and parameter settings. Measures of accuracy, AUC, brier score, L1 error, cross-entropy, and ROC surface plots are used as evaluation criteria comparing the priors with frequentist methods as Lasso, Elastic-Net, and Ridge regression.

**Results:**

All three priors can be used for robust prediction on significant metrics, irrespective of their categorical response model choices. Simulation studies could achieve the mean prediction accuracy of 91.6% (95% CI: 88.5, 94.7) and 76.5% (95% CI: 69.3, 83.8) for logistic regression and multinomial logistic models, respectively. The model can identify significant variables for disease risk prediction and is computationally efficient.

**Conclusions:**

The models are robust enough to conduct both variable selection and prediction because of their high shrinkage properties and applicability to a broad range of classification problems.

**Supplementary Information:**

The online version contains supplementary material available at (10.1186/s12874-022-01560-6).

## Introduction

Recent innovations and availability of structured electronic health records (EHRs), and multisite longitudinal studies, high-throughput sequencing (HTS) [1] data have made patient characteristics, genomics information accessible for statistical prediction analysis and clinical research. Medical decisions are modified upon the identification of essential covariates for a specific clinical outcome. Treatment recommendations are also age, population, and comorbidity specific, with HTS bringing a paradigm shift in large-scale personalized medicine. For example, in diabetes prediction, various biological and clinical factors such as sex, age, obesity are responsible for predicting the future risk of the disease. In oncology, for instance, in colorectal cancer [2], breast cancer [3], non-small cell lung cancer(NSCLS) prognostic [4], and predictive genes such as human epidermal growth factor receptor 2 (HER2), BRAF, KRAS are not only essential for early detection but also therapy selection, subgroup stratification, and controlled monitoring of the disease. In Alzheimer’s disease (AD), a combination of traditional risk factors including age, education, hypertension, obesity, cognitive test scores, cardiovascular disease [5] along with testing of ApoE4 gene can be informative in identifying if a patient is vulnerable to the non-curable disease or any other forms of dementia.

A clinical prediction or classification model, followed by a variable selection strategy, plays a vital role in designing preventive measures from adverse outcomes.

### Penalized regression

Variable selection methods can mitigate noise imposed by irrelevant variables in the data. Most of these methods are frequentist approaches such as Lasso [6], Elastic-Net [7] that uses L1 or L2 penalty to shrink the coefficients of irrelevant variables. Bayesian regression methods that incorporates shrinkage properties as prior information is a reasonable alternative approach, and is the ultimate focus of this research work.

Public health researchers often encounters prediction problems with categorical responses. Depending on the clinical features the primary outcome needs to be predicted, such as the classification of a Covid-19 patient as “discharged” or “died” or “remained in hospital” with individual-patient level data [8]. When the response variable is binary, logistic regression (LR) models enable assessing the association between an independent variable(s) and the response variable. When the response variable has more than two categories, generalizations of the logistic model, such as multinomial logistic regression (MLR) models, are common. The above example provides a better understanding of categorical response models and their contribution as a natural and attractive approach in a diverse array of applications, ranging from spam detection, credit card fraud to predicting Alzheimer’s stage and tumor malignancy. The categorical response models’ primary goal is to infer relationships between a categorical response and a set of explanatory variables. As in any form of regression technique, this is critical to understanding the causal dynamics of a complex system and making accurate predictions on future data. Depending on a specific problem, the explanatory variables or predictors can be of the form of demographic profiles, socio-economic data, or complex biomolecular entities. Modeling becomes particularly challenging when the number of such predictors grows large, a common feature in genomics. Traditionally, frequentist likelihood-based methods have been using penalized regression techniques to address this “curse of dimensionality” and to deal with data having a considerable number of predictors in linear and multicategory response regression. These methods briefly add penalty terms to the negative log-likelihood function and then optimize this penalized likelihood for parameter inference. The penalization leads to a desired “shrinking” behavior of the estimates. Specifically, it pulls the small coefficients towards zero while minimally affecting the large coefficients, thus selecting only the most relevant variables. For a LR set up, the objective function that needs to be minimized is of the form

1 $$l_{\lambda}^{LR}(\beta)=\sum_{i=1}^{n}y_{i} x_{i} \beta-\log(1+e^{x_{i}^{T} \beta})+\lambda\left(\sum_{j=1}^{p}|\beta_{j}|^{r}\right)^{\frac{1}{r}},r>0.$$

Here, *y*=(*y*_1_,*y*_2_,...,*y*_*n*_) is an *n*- dimensional vector representing the outcome variable; *x*_*i*_=(*x*_*i*1_,*x*_*i*2_,…,*x*_*ip*_) are the covariates with *x*_*ij*_ are the observed values on the *p* predictors and (*β*_1_,*β*_2_,...,*β*_*p*_)^*T*^ is a *p*-dimensional parameter vector of regression coefficients. *λ* is the penalty parameter which modulates the amount of shrinkage. Large values of *λ* lead to more shrinkage, while *λ*=0 leads to the simple logistic likelihood without shrinkage. The generalized objective function for multiple categories is

2 $$l_{\lambda}^{MLR}(\beta)=\sum_{i=1}^{N} y_{i} (\sum_{k=0}^{K}x_{ik} \beta_{k}-n_{i} \log(1+e^{\sum_{k=0}^{K}{x_{ik} \beta_{k}}})+\lambda\left(\sum_{k=1}^{K}\sum_{j=1}^{p}|\beta_{kj}|^{r}\right)^{\frac{1}{r}}.$$

Different values of *r* lead to various penalization techniques. For example, *r*=1 results in the well-known Least Absolute Shrinkage and Selection Operator (Lasso) [6] solution and *r*=2 results in the Ridge regression solution [9]. Elastic-Net [7] is another method that is a convex combination of Lasso and Ridge.

### Shrinkage priors

Bayesian variable selection models form a natural counterpart to penalization, with some specialized priors assuming the penalty terms’ role. Some notable works in this area include [6, 10–18] among many. A comprehensive overview of shrinkage priors for data applications is present in [19]. The historical development of shrinkage priors can be traced back to the spike and slab approach proposed in [20]. It is a hierarchical mixture model that essentially uses a latent binary indicator to differentiate between the large and small regression coefficients. It does so by assigning Gaussian priors with high and low variances to the coefficients, conditional on these indicators. The resultant marginal prior for a coefficient takes on a shape with a “spike” at 0 and a relatively flat “slab” at non-zero regions, lending the model its name. Stochastic search variable selection (SSVS) [21] is used for identifying a subset of significant covariates with a hierarchical normal mixture model structure, similar to the spike and slab prior. However, it uses continuous Gaussian distribution to include and exclude variables and lower variance, respectively. The spike-and slab prior has a bi-separation effect on the model coefficients, thus bringing a computational complexity of 2^*p*^. SSVS is computationally intensive and cannot tackle a large set of predictors. Inclusions of non-conjugate and conjugate priors are detailed in this extension of the work [22]. A related class of variable selection (VS) models put the positive prior mass at 0. Inference in these models usually relies on Reversible Jump sampling techniques [23]. Though these models have the nice property of explicitly reducing coefficients to zero, they typically incur high computational costs and exhibit poor mixing. These issues necessitated using a computationally efficient class of priors, which allowed sufficient shrinkage in high dimensions. The focus solely is on such priors for this article. These priors are called global-local(GL) shrinkage priors [24]. Denoting the parameter set as *β*=(*β*_1_,...,*β*_*p*_)^*T*^, the GL framework can be written hierarchically as follows

*β*_*j*_∼*N*(0,*λ*_*j*_*τ*) *λ*_*j*_∼*f*(.) and *τ*∼*g*(.).

The global parameter *τ* controls the overall shrinkage of all the coefficients towards zero while the local parameters *λ*=(*λ*_1_,...,*λ*_*p*_) modulate the coefficient-specific shrinkage. It is desired that the distribution of the local parameter *f*(.) has heavy tails and the distribution of the global parameter *g*(.) has substantial mass at point zero. The Normal-Gamma prior [25], the Dirichlet Laplace (DL) prior [18], Horseshoe prior [26], and the Double Pareto (DP) prior [17] are some of the GL priors. A review and comparison of shrinkage methods for VS are detailed in [27]. High-dimensional statistical modelling with Bayesian shrinkage prior method has been extended in several arenas such as longitudinal binary data with informative missingness [28], and for joint modelling of clustered mixed outcomes with uniform shrinkage prior [29]. The second piece of our proposed approach focuses on a convenient data augmentation representation of the logistic regression likelihood. Two data augmentation algorithms that have gained popularity for binary responses which are for the probit regression model that uses truncated normal random variables [30] and another for the logistic regression that uses Polya-Gamma (PG) random variables [31]. Here we focus on the latter scheme.

In the following sections, the PG Data Augmentation is described under LR and MLR models. Next, the data-augmented likelihood framework’s connection to the Horseshoe, DL, and DP priors is presented, embedding them in a fully Bayesian hierarchical model. Performance metrics evaluate the simulation results with different sample sizes, covariate dimensions, and parameter settings with several data applications. The limitations and contributions are in the Discussion section where the article is concluded with future research directions.

## Bayesian binary regression and polya-Gamma data augmentation

Consider the binary logistic regression model where *Y*_1_,*Y*_2_,...,*Y*_*n*_ are i.i.d. Bernoulli random variables with variables $\left\{x_{i}\in \mathbb{R}^{p}\right\}_{i=1}^{n}$ as the covariates and $\beta \in \mathbb {R}^{p}$ denotes the unknown regression coefficients. The corresponding likelihood is given by

3 $$\prod_{i=1}^{n}P(Y_{i} = y_{i}\mid \beta)=\prod_{i=1}^{n} \frac{\exp(x_{i}^{T}\beta y_{i})}{1+\exp(x_{i}^{T}\beta)}.$$

In the context of the Bayesian analysis, the posterior of *β* given the data is given by $\pi (\beta \mid Y) \propto \pi (\beta)\prod _{i=1}^{n} \frac {\exp (x_{i}^{T}\beta y_{i})}{1+\exp (x_{i}^{T}\beta)}$, where *π*(*β*) denotes the prior distribution for *β*. Unfortunately, the above posterior becomes intractable due to the presence of the term $(1+\exp (x_{i}^{T}\beta))$ in its denominator [32]. Therefore, posterior sampling in this setup traditionally relied on the Metropolis Hastings (MH) algorithm. As an alternative, the Data Augmentation (DA) algorithm utilizes the latent variables to circumvent the difficulty. Before introducing to the specific Polya-Gamma DA scheme that we used in this article, we provide the general structure of a DA algorithm. Suppose, we need to sample a parameter of interest *θ* from an intractable density *π*(*θ*). The technique requires to design a suitable joint density *π*(*θ*,*W*) in such a way that it satisfies the following two criteria ; firstly, $\int \pi (\theta, W) dW = \pi (\theta)$ and secondly, the corresponding conditional distributions, *π*(*θ*∣*W*) and *π*(*W*∣*θ*) are possible to sample from [33, 34]. In the context of the Bayesian analysis, *π*(*θ*) typically refers to a posterior density for a parameter of interest *θ*. A major challenge for designing a DA is to construct an appropriate choice for *π*(*θ*,*W*) (see [30], [31]). A commonly used strategy is to build a conditional distribution *π*(*W*∣*θ*) so that *π*(*θ*,*W*)=*π*(*W*∣*θ*)*π*(*θ*) fulfills the requirements. The latent variable *W* is often termed as the augmented random variable. In the current context, our parameter of interest is *β* with the posterior density *π*(*β*∣*Y*) while we make use of the DA technique designed in [31] where the authors develops and utilizes the Polya-Gamma (PG) distribution as the choice for the augmented random variable. As we will require in the later sections, we include the probability density function of the Polya-Gamma distribution (denoted by *PG*(1,*c*)) [31], as following

4 $$\begin{array}{@{}rcl@{}} p(x\mid c)& =& \cosh\left(\frac{c}{2}\right)\exp\left(-\frac{c^{2}x}{2}\right) h(x) \text{ where }\\ h(w)& = & \sum_{k=0}^{\infty}(-1)^{k}\frac{2k+1}{\sqrt{2 \pi w}}\exp\left(-\frac{(2k+1)^{2}}{8w}\right),0< w<\infty. \end{array}$$

To deal with the intractability in Eq. 3, the DA scheme augments independent latent variables, $\{W_{i}\}_{i=1}^{n}$ where $W_{i} \sim PG(1,|x_{i}^{T}\beta |)$. $\{W_{i}\}_{i=1}^{n}$ are also assumed to be independent of $\{Y_{i}\}_{i=1}^{n}$ for *i*=1,…*n*. Then, the joint posterior $\pi (\beta, \{W_{i}\}_{i=1}^{n}\mid \{Y_{i}\}_{i=1}^{n})$ satisfies the two criteria related to a generic DA algorithm that are mentioned above. The integrability criterion trivially holds whereas the the random variables *W*_1_,…,*W*_*n*_ given $\{Y_{i}\}_{i=1}^{n}, \beta$ are independent and follows PG distribution [31]. The posterior conditional for $\beta \mid \{Y_{i}\}_{i=1}^{n}, \{W_{i}\}_{i=1}^{n}$ becomes to be the multivariate normal if the multivariate normal prior is used for *β* [31]. The appearance of normal distribution as a posterior for *β* is a key feature which provides a way to utilize more nontrivial priors for *β* without much difficulty. Specifically, in this manuscript we use the Global-Local priors that we discuss in details in the next section. As mentioned earlier, Bayesian regression for binary responses has been recognized as a hard problem due to the likelihood’s unwieldy form. There have been several efforts towards an improved version of the DA algorithm [30] for probit regression. Some notable works include [35] and [36]. However, these algorithms are more imprecise versions of [30] making it significantly difficult with multiple layers of latent variables and restricting its usage. In contrast, the DA algorithm by [31] is free of these problems and computationally much less cumbersome.

### Logistic regression model with hierarchical prior structures

In this subsection, we include the details of each of the three priors distributions along with the corresponding posterior distributions. The original form of the horseshoe prior [37] is represented as

5 $$\begin{array}{@{}rcl@{}} \\ \beta_{j}\mid\Lambda_{j}^{2}\tau^{2} \sim N_{p}\left(0,\Lambda_{j}^{2}\tau^{2}\right), j=1,2,...,p; \Lambda_{j}^{2} \sim C^{+}(0,1); \tau \sim C^{+}(0,1). \end{array}$$

Another computationally feasible hierarchical representation of the Horseshoe prior [14] that is used here is

6 $$\begin{array}{@{}rcl@{}} \\ \beta_{j}\mid\Lambda_{j}^{2}\tau^{2} \sim N_{p}\left(0,\Lambda_{j}^{2}\tau^{2}\right), j=1,2,...,p; \Lambda_{j}^{2}\mid\gamma_{j} \sim IG\left(\frac{1}{2},\frac{1}{\gamma_{j}}\right); \\ \tau^{2}\mid\xi\sim IG\left(\frac{1}{2},\frac{1}{\xi}\right); \gamma_{1},\gamma_{2},...,\gamma_{p},\xi\sim IG\left(\frac{1}{2},1\right). \end{array}$$

Here, the variance covariance matrix *Σ* of the distribution of *β* is a diagonal matrix with elements (*Λ*_1_*τ*^2^,…*Λ*_*p*_*τ*^2^). From Eqs. 3,(4) and the above hierarchical prior structure (6), the full posterior distribution is given by:

7 $$\begin{array}{@{}rcl@{}} \pi\left(\beta,\Lambda_{j}^{2},w_{i},\gamma_{j},\tau^{2},\xi\mid Y\right) \propto \prod_{i=1}^{n} \frac{\exp(x_{i}^{T}\beta y_{i})}{(1+\exp(x_{i}^{T}\beta))}\left(1+\exp\left(x_{i}^{T}\beta\right)\right) h\left(w_{i}\right) \\ \exp\left(-\frac{x_{i}^{T}\beta}{2}-\frac{\left(x_{i}^{T}\beta\right)^{2}w_{i}}{2}\right) \frac{\exp(-\frac{1}{2}(\beta^{T}\Sigma^{-1}\beta))}{\sqrt{2\pi\Sigma}} \\ \prod_{j=1}^{p}\frac{\left(\Lambda_{j}^{2}\right)^{-(\frac{1}{2}+1)}\exp\left(\frac{-1}{\gamma_{j}\Lambda_{j}^{2}}\right)}{\gamma_{j}^{\frac{1}{2}}}\ \frac{(\tau^{2})^{-\left(\frac{1}{2}+1\right)}\exp\left(\frac{-1}{\tau^{2}\xi}\right)}{\xi^{\frac{1}{2}}} \\ \gamma_{j}^{-(\frac{1}{2}+1)}\exp\left(\frac{-1}{\gamma_{j}}\right)\ \xi^{-(\frac{1}{2}+1)}\exp\left(\frac{-1}{\xi}\right). \end{array}$$

where, *h*(*w*_*i*_) is defined in Eq. 4. The conditional distributions required for the analysis are as follows:

The conditional density of *β* given *y*, *w* is

8 $$\pi\left(\beta\mid \Sigma,W_{D},Y\right)\sim N_{p}\left(\left(X^{T}W_{D}X+\Sigma^{-1}\right)^{-1}X^{T}y^{*},\left(X^{T}W_{D}X+\Sigma^{-1}\right)^{-1}\right)$$

where, *W*_*D*_ and *Σ* are diagonal matrices where the elements are (*w*_1_,*w*_2_,...,*w*_*n*_), $(\Lambda _{1}^{2}\tau ^{2},...,\Lambda _{p}^{2}\tau ^{2})$ respectively and, $y^{*}=\left (y_{1}-\frac {1}{2},....,y_{n}-\frac {1}{2}\right)$.

In high-dimensional scenarios where sampling of *β* can be difficult for *p*>*n* case, fast sampling technique with Gaussian scale-mixture priors [38] is used where the mean and variance of a Gaussian distribution is in the respective form: $N_{p}(\mu,\hat {\Sigma }), \mu =\hat {\Sigma } A^{T}\alpha, \hat {\Sigma }=(A^{T}A+D^{-1})^{-1}$ *D*∈*R*^*p*×*p*^,*A*∈*R*^*n*×*p*^,*α*∈*R*^*n*×1^ The algorithm involves sampling *u*∼*N*(0,*D*), and *δ*∼*N*(0,*I*_*n*_); with *V*=*Au*+*δ* getting the inverse of (*ADA*^*T*^+*I*_*n*_)*w*=(*α*−*v*), and finally obtaining *θ*=*u*+*DA*^*T*^*w*,*θ*∼*N*(*μ*,*Σ*). The conditional density of *w*_*i*_ given *x*_*i*_,*β* is

9 $$\begin{array}{@{}rcl@{}} \pi\left(w_{i}\mid\beta\right)\sim PG\left(1,x_{i}^{T}\beta\right). \end{array}$$

The conditional density of the hyper-parameters are as follows

10 $$\begin{array}{@{}rcl@{}} \pi(\Lambda_{j}^{2}\mid \gamma_{j},\beta_{j},\xi,\Lambda_{j}^{2})\sim IG\left(1,\frac{1}{\gamma_{j}}+\frac{\beta_{j}^{2}}{2\tau^{2}}\right) \\ \pi(\gamma_{j}\mid \Lambda_{j}^{2},\beta_{j},\xi,\tau^{2})\sim IG\left(1,1+\frac{1}{\Lambda_{j}^{2}}\right) \\ \pi(\tau^{2}\mid \gamma_{j},\beta_{j},\xi,\Lambda_{j}^{2})\sim IG\left(\frac{p+1}{2},\frac{1}{\xi}+\sum_{j=1}^{p}\frac{\beta_{j}^{2}}{2\Lambda_{j}^{2}}\right) \\ \pi\left(\xi\mid \tau^{2}\right)\sim IG\left(1,1+\frac{1}{\tau^{2}}\right). \end{array}$$

Here, all the posterior densities are in the closed form, and follow simple probability densities such as Normal, Polya-Gamma and Inverse-Gamma making sampling from them trivial. The hierarchical structure of the Dirichlet Laplace prior [18] is

11 $$\begin{array}{@{}rcl@{}} \beta_{j} \sim N_{p}\left(0,\psi_{j}\phi_{j}^{2}\tau^{2}\right); \psi_{j} \sim exp\left(\frac{1}{2}\right); \phi \sim Dir\left(a,a,...,a\right); \tau \sim G\left(pa, \frac{1}{2}\right). \end{array}$$

The conditional posterior distributions remain same for *β*∣*y*_*i*_ and *w*_*i*_∣*β* is similar to that of Eqs. 8 and 9.

The conditional density of the hyper-parameters as obtained similar to Theorem 2.2 in [18] are as follows:

12 $$\begin{array}{@{}rcl@{}} \pi(\psi\mid \phi,\tau,\beta)\sim IG\left(\frac{\phi_{j}\tau}{|\beta_{j}|},1\right) \\ \pi(\tau\mid \phi,\beta)\sim GIG\left(pa-p,1,2\sum_{j=1}^{p}\frac{|\beta_{j}|}{\phi_{j}}\right). \end{array}$$

To sample *π*(*ϕ*∣*β*_*j*_) sample *T*_*j*_∼*GIG*(*a*−1,1,2|*β*_*j*_|), set $\phi _{j} =\frac {T_{j}}{T}, T=\sum _{j=1}^{p}T_{j}$. where *GIG*(*a*,*b*,*c*) is the Generalized Inverse Gaussian distribution with density $f(x; a, b,c) \propto x^{(c-1)} e^{\frac {-1}{2}(ax+\frac {b}{x})}$.

The hierarchical structure of Double Pareto prior[30] is

13 $$\begin{array}{@{}rcl@{}} \\ \beta\mid\Lambda,\tau \sim N_{p}(0,D_{\tau}); w_{i}\mid x_{i},\beta \sim PG\left(1,x_{i}^{T}\beta\right); \\ \tau_{j}\mid\Lambda_{j} \sim exp\left(\frac{\Lambda^{2}}{2}\right); \Lambda_{j} \sim G(\zeta,\eta). \end{array}$$

Again, the conditional densities of *β*∣*y*_*i*_ and *w*_*i*_∣*β* remains same as (8) and (9). Here *Σ*=*D*_*τ*_ is a diagonal matrix with elements (*τ*_1_,*τ*_2_,...,*τ*_*p*_). The conditional density of rest of the hyper-parameters are as follows:

14 $$\begin{array}{@{}rcl@{}} \pi\left(\tau_{j}\mid \beta, \Lambda,y\right)\sim GIG\left(\frac{1}{2}, \Lambda_{j}^{2}, \beta_{j}^{2}\right)\\ \pi\left(\Lambda\mid\beta,y\right)\sim Gamma\left(\zeta +1, \eta+ \left|\beta_{j}\right|\right). \end{array}$$

### Extending hierarchical models and differential shrinkage

The strength of our methods are in no way limited to a common shrinkage prior across covariates. In fact, this could be applied to several different variant models which allow for borrowing strength across multiple responses and different (in some cases, user-defined) levels of sparsity among groups of covariates. In particular, this could be used for models where we can choose not to shrink some covariates (for instance, some demographic covariates) in contrast to the genomic covariates.

In terms of estimation procedures, the hierarchical structure of the models would allow the form of the posterior conditional of *β* would remain the same in the model variants. Only the diagonal weight matrices appearing in the posterior means and variances would have some differential allocation of *λ*s and constant variances depending on which variable that we choose to shrink. Similarly, the posterior sampling of the shrinkage hyper-parameters would be a subset. Details of the derivation are omitted since these are straight forward.

## Bayesian multinomial logistic regression and polya-Gamma data augmentation

This is the extension of the binary regression, where the outcome variable has more than two classes. Let *Y*_*i*_, *i*=1,2,…,*n* be a categorical random variable with *k* categories, where *k*≥2. The probability for the *k*^*th*^ category is *p*_*ik*_=*P*(*Y*_*i*_=*k*∣*x*_*i*_), where $\sum _{k=1}^{K} p_{ik} = 1$. The multinomial logistic regression (MLR) model is given as

15 $$\begin{array}{@{}rcl@{}} \\ P(Y_{i} = k \mid x_{i}) &= p_{ik} &= \frac{e^{x_{i}^{T}\beta_{k}}}{1+\sum_{j=1}^{K-1} e^{x_{i}^{T}\beta_{j}}}, k = 1,2,\ldots K-1 \\ P(Y_{i} = K \mid x_{i}) &= p_{iK} &= \frac{1}{1+\sum_{j=1}^{K-1} e^{x_{i}^{T}\beta_{j}}}. \end{array}$$

Here, *β*_*k*_ are the coefficients associated with *k*-th category, and *K* is the baseline category with its coefficient *β*_*K*_ constrained to zero. The Polya-Gamma data augmentation approach can be extended for multi-category response variables. Here, *β*_*k*_ is updated conditional on the remaining *β*_−*k*_=(*β*_1_,*β*_2_,…,*β*_*k*−1_,*β*_*k*+1_,…,*β*_*K*_). The full conditional for *β*_*k*_ given *y* and *β*_*j*≠*k*_ can be expressed as a likelihood of the Bernoulli distribution.

16 $$f(\beta_{k} \mid y, \beta_{-k}) \propto f(\beta_{k}) \Pi_{i=1}^{n} p_{ik}^{I(y_{i}=k)}(1-p_{ik})^{1-I(y_{i}=k)}$$

where *f*(*β*_*k*_) is the prior for coefficient *β*_*k*_,*I*(*Y*_*i*_=*k*)=1 when *Y*_*i*_=*k* with probability $p_{ik} = P(Y_{i}=k)= \frac {e^{x_{i}^{T}\beta _{k}}}{\sum _{j=1}^{K} e^{x_{i}^{T}\beta _{k}}}$. It can be re-written as $p_{ik} = P(Y_{i}=k) = \frac {e^{x_{i}^{T}\beta _{k}-M_{ik}}}{1+ e^{x_{i}^{T}\beta _{k}-M_{ik}}} = \frac {e^{\psi _{ik}}}{1+e^{\psi _{ik}}}$ where, $M_{ik}= \log \sum _{j \neq k}e^{x_{i}^{T}\beta _{j}}$ and $\psi _{ik} = x_{i}^{T}\beta _{k}-M_{ik}$. $\sum _{j \neq k}e^{x_{i}^{T}\beta _{j}}$ includes the reference category *K*, as *β*_*K*_=0, so $e^{x_{i}^{T}\beta _{k}} =1$, and hence $M_{ik} = \log \sum _{j \neq k}e^{x_{i}^{T}\beta _{j}} = \log \left (1+\sum _{j \neq {k,K}}e^{x_{i}^{T}\beta _{j}}\right)$. Thus, the full conditional for *β*_*k*_ given *y* and *β*_−*k*_ is

17 $$\begin{array}{@{}rcl@{}} f(\beta_{k}\mid y,\beta_{-k}) \propto f(\beta_{k}) \Pi_{i=1}^{n} \frac{(e^{\psi_{ik}})^{I(y_{i}=k)}}{1+e^{\psi_{ik}}} \end{array}$$

which is a logistic regression likelihood, thus PG data augmentation can be implemented to update each of *β*_*k*_’s based on the binary indicator variable *I*(*y*_*i*_=*k*). The above Eq. 17 after including PG data augmentation and prior for *β*_*k*_∼*N*(0,*Σ*_*k*_) can be expressed as:

18 $$\begin{array}{@{}rcl@{}} f(\beta_{k}\mid y,\beta_{-k}) = \Pi_{i=1}^{n} \frac{(e^{\psi_{ik}})^{I(y_{i}=k)}}{1+e^{\psi_{ik}}} (1+e^{\psi_{ik}}) e^{-\psi_{ik}/2-\psi_{ik}^{2}w_{ik}/2}\\ h(w_{ik}) e^{-\frac{1}{2}\left(\beta_{k}^{T}\Sigma_{k}^{-1}\beta_{k}\right)}. \end{array}$$

### Multinomial logistic regression with hierarchical prior structures

The hierarchical structure with three priors was calculated similarly to LR. Considering $N(0,\Lambda _{t}^{2}\tau ^{2}), t = 1,2,\ldots,p$, for *β*_1_,*β*_2_,…,*β*_*K*−1_, the conditional density for *β*_*k*_ and *w*_*ik*_ are given as

19 $$\begin{array}{@{}rcl@{}} \beta_{k} \sim N(\mu_{k},\Sigma_{k}),\ \mu_{k} = \Sigma_{k} \left(\Sigma_{0} + X^{T}W_{k} y_{k}^{*}\right), \ \Sigma_{k} = \left(\Sigma_{0}+X^{T}W_{k}X\right)^{-1}\\ w_{ik} \sim PG(1, \psi_{ik}). \end{array}$$

where $$\Sigma _{0} =diag\left (\Lambda _{t}^{2}\tau ^{2}\right), W_{k} = diag(w_{ik}), y_{k}^{*} = \frac {I(y_{i}=k)-0.5}{w_{ik}}+M_{ik}$$.

The conditional density of the hyper-parameters in the case of horseshoe prior is

20 $$\begin{array}{@{}rcl@{}} \pi(\Lambda_{jk}^{2}\mid \gamma_{jk},\beta_{jk},\xi,\Lambda_{jk}^{2})\sim IG\left(1,\frac{1}{\gamma_{jk}}+\frac{\beta_{jk}^{2}}{2\tau^{2}}\right) \\ \pi(\gamma_{jk}\mid \Lambda_{jk}^{2},\beta_{jk},\xi,\tau^{2})\sim IG\left(1,1+\frac{1}{\Lambda_{jk}^{2}}\right) \\ \pi(\tau^{2}\mid \gamma_{jk},\beta_{jk},\xi,\Lambda_{jk}^{2})\sim IG\left(\frac{p+1}{2},\frac{1}{\xi}+\sum_{t=1}^{p}\frac{\beta_{jk}^{2}}{2\Lambda_{jk}^{2}}\right) \\ \pi\left(\xi\mid \tau^{2}\right)\sim IG\left(1,1+\frac{1}{\tau^{2}}\right) \end{array}$$

The conditional density of the hyper-parameters in case of DL prior is

21 $$\begin{array}{@{}rcl@{}} \pi(\psi\mid \phi,\tau,\beta_{k})\sim IG\left(\frac{\phi_{tk}\tau}{|\beta_{tk}|},1\right) \\ \pi(\tau\mid \phi,\beta_{k})\sim GIG\left(pa-p,1,2\sum_{j=1}^{p}\frac{|\beta_{tk}|}{\phi_{tk}}\right). \end{array}$$

To sample *π*(*ϕ*∣*β*_*k*_) sample *T*_*tk*_∼*GIG*(*a*−1,1,2|*β*_*tk*_|), set $\phi _{tk} =\frac {T_{tk}}{T}, T=\sum _{t=1}^{p}T_{tk}$. where *GIG*(*a*,*b*,*c*) is the Generalized Inverse Gaussian distribution with density $f(x; a, b,c) \propto x^{(c-1)} e^{\frac {-1}{2}(ax+\frac {b}{x})}$.

The conditional densities of the hyper-parameters are as follows:

22 $$\begin{array}{@{}rcl@{}} \pi\left(\tau_{tk}\mid \beta_{k}, \Lambda,y\right)\sim GIG\left(\frac{1}{2}, \Lambda_{tk}^{2}, \beta_{tk}^{2}\right)\\ \pi\left(\Lambda\mid\beta_{k},y\right)\sim Gamma\left(\zeta +1, \eta+ \left|\beta_{tk}\right|\right). \end{array}$$

Here, *β*_*k*_=(*β*_1*k*_,*β*_2*k*_,…,*β*_*pk*_),*t*=1,2,…,*p*.

## Simulation and results

Data was simulated from the logistic regression model under various settings of sample size (*n*), dimensions (*p*), and effect sizes *β*=(*β*_1_,*β*_2_,…,*β*_*p*_). The continuous covariates are simulated with mean *m* and *sd*=1. A total of 100 data sets were simulated for each of the conditions. All computations are carried out in RStudio [39]. The length of the Markov Chain Monte Carlo (MCMC) simulation is 10000, and 6000 iterations are discarded in the burn-in step. The value of parameter *a* in DL prior is chosen as 0.8 for optimal results and convergence.

### Post-processing of mCMC samples

After model fitting variable selection was implemented by computing their posterior credible intervals. Posterior credible intervals can be readily obtained from a Bayesian framework via MCMC samples, and can provide direct uncertainty measures irrespective of model complexity. Specifically, these intervals are constructed by the quantiles of the MCMC samples of the parameters and estimate the probability intervals of the true posterior distribution. The variables are declared to be significant if and only if these posterior credible interval do not include zero. Thus, it will be a binary vector of the variables that were selected from estimation. These binary estimates were then matched against the simulation truth to compute some performance metrics.

Other than performance measures, these credible interval techniques would also provide a convenient measure of uncertainty, in case we need to use them. The varying sizes and noise content of datasets gets reflected in the varying lengths of the estimated prediction intervals that in turn allows us to place varying degrees of confidence in our inferred relationships. This could also inform us about the desired adequacy of sample size and number of variables for future studies. This can be readily obtained from MCMC samples unlike other traditional methods such as Lasso.

### Performance measures

Based on the binary estimates, sensitivity, specificity and overall accuracy were computed for variable selection. In addition, two continuous error measures, MSE and Brier scores,were computed. The Brier Score (BS) is defined as $BS= \frac {1}{N}\sum _{i=1}^{N} \left (P_{i} -Y_{i}\right)$; here, *P*_*i*_ is the probability of prediction and *Y*_*i*_ is the actual outcome at that instance. The best score achievable is 0 and the worst is 1; The Mean Squared Error (MSE) is given by ${\frac {1}{p}\sum _{i=1}^{p}(\beta _{i}-\hat {\beta _{i}})^{2}}$;

### Prediction

A prediction module was also added that would assess how the model fares in classifying the responses. For this, a cross-validation approach and divided each simulated data set into training and test data on a 80-20 allocation percentage. The following measures were used for assessing the quality of our prediction. Accuracy (the proportion of correctly classified responses in the test dataset); Sensitivity (proportion of true positives); Specificity (proportion of true negatives); AUC (Area Under the Receiver Operating Characteristic (ROC) Curve).

### Simulation scenarios and tables

Table 1 summarizes the simulation results and simulation settings for prediction and VS and Table 2 records the same for MLR.

**Table 1:** Prediction & Variable Selection Performance for LR with Shrinkage Priors

	Prediction								
Priors	BS1	BS2	BS3	BS4	BS5	BS6	BS7	BS8	BS9
N,P, *ρ*	1000,10,0.5	200,10,0.5	400,20,0.5	500,50,0.3	300,10,0.5	100,10,0.5	100,130,0.5	1000,10,0	1000,10,0
*β*	(10,10,10,10,5,5,0.1,0.1,0.1,0.1)’	(10,10,10,10,5,5,0.1,0.1,0.1,0.1)’	$$\left (\begin {array}{l}{\underbrace {10,\ldots,10}_{5}},{\underbrace {5,\ldots,5}_{5}}, {\underbrace {0.1,\ldots,0.1}_{10}}\end {array}\right)$$	40% non-zero *β*	(1,1.5,-2,2.5,0,0,0,0,0,0)’	(5,5,3,0.74,-0.9,0,0,0,0,0)’	$$\left (\begin {array}{l}{\underbrace {5,\ldots,5}_{30}},{\underbrace {0,\ldots,5}_{100}}\end {array}\right)$$	*β*=(3.5,0.2,0.1,1,2,3,5,5,5,5)	*β*=(3.5,3.5,3.5,3.5,5,5,0.1,0.1,0.1,0.1)
	Accuracy								
Horseshoe	0.968(0.012)	0.958(0.031)	0.965(0.021)	0.856(0.032)	0.843(0.048)	0.894(0.074)	0.954(0.053)	0.909(0.018)	0.899(0.024)
Dirichlet Laplace	0.968(0.012)	0.958(0.030)	0.965(0.020)	0.839(0.039)	0.842(0.050)	0.894(0.075)	0.938(0.062)	0.911(0.018)	0.898(0.024)
Double Pareto	0.968(0.012)	0.958(0.031)	0.964(0.021)	0.834(0.040)	0.839(0.050)	0.894(0.073)	0.940(0.050)	0.912(0.018)	0.898(0.024)
	Sensitivity								
Horseshoe	0.967(0.016)	0.965(0.043)	0.964(0.031)	0.853(0.047)	0.840(0.067)	0.897(0.107)	0.940(0.076)	0.909(0.027)	0.914(0.030)
Dirichlet Laplace	0.967(0.016)	0.964(0.043)	0.964(0.031)	0.840(0.053)	0.837(0.068)	0.898(0.106)	0.944(0.073)	0.910(0.028)	0.914(0.030)
Double Pareto	0.967(0.016)	0.964(0.044)	0.964(0.030)	0.835(0.055)	0.835(0.067)	0.898(0.105)	0.946(0.073)	0.912(0.028)	0.922(0.029)
	Specificity								
Horseshoe	0.969(0.017)	0.953(0.046)	0.966(0.031)	0.859(0.052)	0.847(0.070)	0.893(0.094)	0.943(0.084)	0.910(0.027)	0.877(0.038)
Dirichlet Laplace	0.969(0.017)	0.955(0.046)	0.967(0.028)	0.838(0.058)	0.847(0.070)	0.890(0.097)	0.935(0.079)	0.911(0.026)	0.875(0.039)
Double Pareto	0.969(0.017)	0.954(0.045)	0.964(0.030)	0.833(0.058)	0.847(0.070)	0.893(0.094)	0.934(0.079)	0.912(0.027)	0.863(0.041)
	Area Under Curve								
Horseshoe	0.968(0.012)	0.958(0.031)	0.965(0.021)	0.856(0.032)	0.844(0.049)	0.898(0.074)	0.944(0.052)	0.910(0.018)	0.896(0.025)
Dirichlet Laplace	0.968(0.012)	0.958(0.029)	0.966(0.020)	0.839(0.039)	0.842(0.050)	0.897(0.075)	0.939(0.063)	0.911(0.018)	0.895(0.025)
Double Pareto	0.968(0.012)	0.958(0.030)	0.964(0.021)	0.834(0.040)	0.849(0.050)	0.897(0.073)	0.941(0.051)	0.912(0.018)	0.897(0.024)
	Brier Score								
Horseshoe	0.023(0.007)	0.030(0.018)	0.025(0.013)	0.103(0.019)	0.110(0.026)	0.073(0.042)	0.046(0.026)	0.067(0.009)	0.072(0.012)
Dirichlet Laplace	0.023(0.007)	0.029(0.016)	0.025(0.011)	0.114(0.022)	0.111(0.026)	0.073(0.041)	0.044(0.026)	0.066(0.009)	0.072(0.012)
Double Pareto	0.023(0.007)	0.030(0.017)	0.025(0.012)	0.117(0.023)	0.112(0.026)	0.073(0.043)	0.045(0.024)	0.063(0.010)	0.072(0.012)
	Variable Selection								
	Accuracy								
Horseshoe	0.989(0.035)	0.923(0.072)	0.922(0.053)	0.999(0.004)	0.980(0.043)	0.827(0.066)	0.422(0.262)	0.747(0.056)	0.868(0.085)
Dirichlet Laplace	0.994(0.024)	0.920(0.070)	0.914(0.049)	0.972(0.024)	0.981(0.044)	0.829(0.064)	0.504(0.275)	0.758(0.062)	0.856(0.107)
Double Pareto	0.985(0.039)	0.927(0.071)	0.926(0.052)	0.947(0.034)	0.977(0.047)	0.832(.071)	0.527(0.274)	0.820(0.065)	0.940(0.078)
	Sensitivity								
Horseshoe	1.000(0.000)	0.885(0.113)	0.860(0.096)	0.998(0.025)	0.962(0.090)	0.662(0.123)	0.163(0.094)	0.689(0.072)	0.820(0.100)
Dirichlet Laplace	1.000(0.000)	0.868(0.114)	0.838(0.093)	0.998(0.025)	0.978(0.072)	0.668(0.114)	0.136(0.096)	0.704(0.079)	0.848(0.106)
Double Pareto	1.000(0.000)	0.882(0.112)	0.864(0.096)	1.000(0.000)	0.978(0.072)	0.682(0.121)	0.129(0.096)	0.785(0.076)	0.978(0.056)
	Specificity								
Horseshoe	0.972(0.086)	0.980(0.068)	0.985(0.039)	0.999(0.004)	0.992(0.037)	0.992(0.039)	0.999(0.004)	0.980(0.098)	0.940(0.113)
Dirichlet Laplace	0.985(0.060)	0.998(0.025)	0.989(0.031)	0.969(0.026)	0.983(0.058)	0.990(0.044)	0.999(0.003)	0.975(0.110)	0.868(0.165)
Double Pareto	0.962(0.096)	0.995(0.035)	0.988(0.033)	0.942(0.037)	0.977(0.050)	0.982(0.058)	0.999(0.002)	0.960(0.136)	0.882(0.165)
	L1 error								
Horseshoe	2.358(0.417)	3.444(3.895)	2.152(1.194)	0.057(0.017)	0.197(0.064)	1.311(1.776)	1.977(0.229)	1.998(0.109)	1.696(0.053)
Dirichlet Laplace	2.474(0.326)	2.953(0.388)	2.148(0.329)	0.187(0.045)	0.213(0.073)	0.644(0.289)	1.955(0.220)	1.970(0.068)	1.689(0.055)
Double Pareto	2.421(0.387)	2.669(0.601)	1.997(0.438)	0.230(0.053)	0.231(0.076)	0.938(0.720)	1.960(0.237)	1.669(0.077)	1.479(0.089)
	L2 error								
Horseshoe	3.063(0.572)	4.435(4.931)	2.971(1.722)	0.102(0.036)	0.259(0.091)	1.942(2.797)	2.562(0.299)	2.384(0.128)	2.128(0.060)
Dirichlet Laplace	3.231(0.446)	3.849(0.507)	3.015(0.585)	0.277(0.081)	0.276(0.103)	0.896(0.456)	2.582(0.293)	2.349(0.075)	2.118(0.063)
Double Pareto	3.146(0.529)	3.469(0.772)	2.781(0.459)	0.330(0.092)	0.293(0.106)	1.359(1.176)	2.594(0.333)	1.980(0.088)	1.855(0.102)

**Table 2:** Prediction Performance & Variable Selection for MLR with Shrinkage Priors

	Prediction						
Priors	MS1	MS2	MS3	MS4	MS5	MS6	MS7
N,P,J,m	400,4, 3, (-2,-1,0,1,2)’	250,4,3,(-2,-1,0,1,2)’	400,10, 3,0	1000,30, 3,0	500,50, 3,0	600,20, 5,0	300,400, 3,0
*β*	(*β*_1_,*β*_2_,*β*_3_)^′^,*β*_1_=(−1.3,1.2,0,0,2)^′^,*β*_2_=(2,−1.5,0,0)^′^,*β*_3_=0	*β*_1_=(−1.3,1.2,0,0,2)^′^,*β*_2_=(2,−1.5,0,0)^′^,*β*_3_=0	*β*_1_ = *β*_2_ = (-1.3,-1.2,-0.5,-0.5,2,0,0,0,0,0)’, *β*_3_=0	*β*_1_=(*A*_7_,*B*_13_,*C*_7_,*D*_3_)^′^,*A*_7_∼*N*(2,0.25),*B*_13_=0,*C*_7_∼*N*(2,0.25),*D*_3_=0.*β*_2_=(*E*_10_,*F*_7_,*G*_13_),*E*_10_=0,*F*_7_∼*N*(2,0.25),*G*_13_=0	*β*∼*N*(0,1)	*β*_1_=(*A*_7_,*B*_13_,*C*_7_,*D*_3_)^′^,*A*_7_∼*N*(2,0.25),*B*_13_=0,*C*_7_∼*N*(2,0.25),*D*_3_=0,*β*_2_=(*E*_10_,*F*_7_,*G*_13_),*E*_10_=0,*F*_7_∼*N*(2,0.25),*G*_13_=0	$$\beta =\left (\begin {array}{l} {\underbrace {N(0,1),\dots,N(0,1)}_{30}},{\underbrace {0,\dots,0}_{170}},\\ {\underbrace {N(0,1),\dots,N(0,1)}_{30}},{\underbrace {0,\dots,0}_{170}} \end {array}\right)$$
	Accuracy						
Horseshoe	0.812(0.048)	0.812(0.047)	0.712(0.078)	0.873(0.022)	0.816(0.044)	0.760(0.034)	0.576(0.071)
Dirichlet Laplace	0.811(0.048)	0.811(0.050)	0.722(0.050)	0.875(0.023)	0.818(0.043)	0.759(0.034)	0.602(0.068)
Double Pareto	0.813(0.048)	0.812(0.046)	0.713(0.078)	0.873(0.021)	0.816(0.044)	0.760(0.034)	0.583(0.069)
	Miss-classification Error						
Horseshoe	0.188(0.048)	0.188(0.047)	0.282(0.052)	0.127(0.022)	0.184(0.044)	0.240(0.034)	0.424(0.071)
Dirichlet Laplace	0.189(0.048)	0.189(0.050)	0.278(0.050)	0.125(0.023)	0.182(0.043)	0.241(0.034)	0.398(0.068)
Double Pareto	0.187(0.048)	0.188(0.046)	0.281(0.052)	0.127(0.021)	0.184(0.044)	0.240(0.034)	0.417(0.069)
	C-Entropy						
Horseshoe	0.487(0.071)	0.491(0.069)	0.670(0.073)	0.327(0.031)	0.432(0.072)	0.632(0.060)	1.557(0.308)
Dirichlet Laplace	0.480(0.089)	0.488(0.097)	0.665(0.100)	0.313(0.056)	0.620(0.177)	0.677(0.101)	4.113(0.969)
Double Pareto	0.488(0.070)	0.492(0.069)	0.671(0.072)	0.330(0.030)	0.430(0.073)	0.634(0.059)	1.963(0.424)
	AUC						
Horseshoe	0.719(0.060)	0.731(0.065)	0.710(0.047)	0.877(0.029)	0.824(0.050)	0.771(0.030)	0.632(0.066)
Dirichlet Laplace	0.708(0.059)	0.713(0.072)	0.716(0.047)	0.884(0.026)	0.827(0.048)	0.767(0.039)	0.654(0.062)
Double Pareto	0.720(0.059)	0.730(0.064)	0.713(0.048)	0.877(0.028)	0.825(0.048)	0.773(0.031)	0.636(0.065)
	Variable Selection						
	Accuracy						
Horseshoe	0.996(0.021)	0.970(0.057)	0.901(0.040)	0.986(0.015)	0.776(0.033)	0.754(0.039)	0.850(0.071)
Dirichlet Laplace	0.969(0.060)	0.934(0.091)	0.929(0.055)	0.954(0.026)	0.812(0.039)	0.789(0.048)	0.880(0.006)
Double Pareto	0.992(0.030)	0.976(0.052)	0.903(0.036)	0.986(0.014)	0.776(0.034)	0.749(0.039)	0.868(0.004)
	Sensitivity						
Horseshoe	0.998(0.025)	0.950(0.101)	0.838(0.056)	1.000(0.000)	0.675(0.045)	0.659(0.057)	0.052(0.021)
Dirichlet Laplace	0.970(0.082)	0.920(0.132)	0.936(0.069)	1.000(0.000)	0.849(0.046)	0.779(0.062)	0.237(0.033)
Double Pareto	0.998(0.025)	0.965(0.087)	0.838(0.053)	1.000(0.000)	0.677(0.047)	0.651(0.055)	0.122(0.026)
	Specificity						
Horseshoe	0.995(0.035)	0.990(0.049)	0.964(0.056)	0.978(0.024)	0.920(0.036)	0.875(0.051)	0.000(0.000)
Dirichlet Laplace	0.968(0.084)	0.948(0.125)	0.922(0.089)	0.929(0.040)	0.759(0.063)	0.803(0.075)	0.993(0.004)
Double Pareto	0.988(0.055)	0.988(0.055)	0.968(0.053)	0.979(0.022)	0.919(0.040)	0.875(0.049)	0.999(0.002)
	L1 error						
Horseshoe	0.255(0.035)	0.294(0.038)	0.275(0.024)	0.404(0.012)	0.335(0.013)	0.323(0.019)	0.006(0.006)
Dirichlet Laplace	0.215(0.077)	0.293(0.113)	0.206(0.060)	0.246(0.047)	0.550(0.035)	0.366(0.055)	0.024(0.024)
Double Pareto	0.260(0.035)	0.295(0.037)	0.281(0.024)	0.414(0.012)	0.328(0.014)	0.332(0.018)	0.011(0.011)
	L2 error						
Horseshoe	0.313(0.035)	0.358(0.037)	0.354(0.023)	0.582(0.009)	0.430(0.013)	0.409(0.018)	0.007(0.007)
Dirichlet Laplace	0.270(0.105)	0.366(0.149)	0.256(0.072)	0.313(0.064)	0.687(0.044)	0.464(0.067)	0.025(0.025)
Double Pareto	0.322(0.035)	0.363(0.037)	0.364(0.025)	0.601(0.010)	0.419(0.015)	0.420(0.017)	0.013(0.013)

**Fig. 1:**
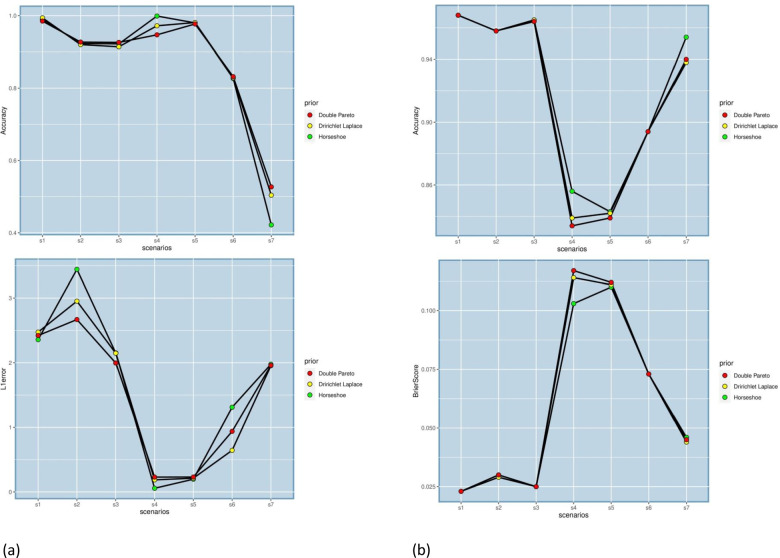
(**a**) Variable Selection and (**b**) Prediction Performance in LR with Shrinkage Priors across Simulation Scenarios

**Fig. 2:**
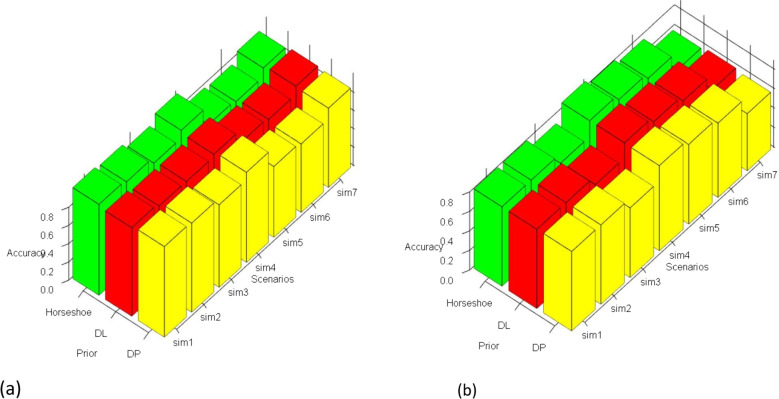
(**a**) Variable Selection and (**b**) Prediction Performance in MLR with Shrinkage Priors across Simulation Scenarios

Each measure has three rows corresponding to the three priors. Figures 1 and 2 represents the VS and prediction performance across the simulation scenarios for LR and MLR, respectively. The two simulation scenarios (BS8 and BS9) reflect the extension discussed in the earlier section– namely, the differential shrinkage option. In BS8, some demographic variables (gender, age, weight, height) as well as their interactions were included with the remaining covariates. The coefficients for the demographic variables and their interactions were not penalized, i.e. given non-shrinkage priors. Another simulation scenario (BS9) was explored that included the same four demographic variables along with four binary variables ∼*Ber*(1,0.2). These proxied SNP variables- (*sn*1,*sn*2,*sn*3,*sn*4)–that arise in genomics applications. Interactions between (*sn*3,*weight*), and (*sn*3,*age*) were considered. Shrinkage was applied to all the main effects of SNPs and the interactions.

The primary takeaway from the results in the tables is that performance metrics reach increasingly higher levels with higher N/P ratio and higher magnitude of the coefficients. In Table 1, for LR, a large part of these numerical measures lie in the range of 0.8-0.9 and the trend also continues in the simulation settings with differential shrinkage and interaction coefficients (BS8 and BS9). Most of the non-zero interaction coefficients in these settings were correctly identified. Higher correlation among the variables, as in BS5, and higher percentage of zero *β*^′^*s* (BS6) does drop the performance to some extent. Overall, the proposed method does markedly better on specificity than sensitivity. Even in the p>n scenario, most of our performance metrics stayed above 0.8 for both prediction and variable selection. In Table 2, for MLR, larger sample size, as before drives better performance. Both large N and large P (MS7), although performing well in variable selection, has the worst predictive performance of all. We intend to explore such settings in more depth in future work with novel shrinkage methods specially geared towards such settings. Overall, performance scores in the range of 0.85-0.9 or above dominate the results in variable selection. The predictive performance, in contrast, is moderate compared to the counterpart in Table 1 for LR, though the prediction accuracies remain above 0.8 in 4 out of the 7 simulation settings. It is seen that a larger number of classes, as in MS6 (5 classes), push down the predictive measures to the range of 0.7-0.8. Lastly, the performances are very similar across all three priors for variable selection as well as prediction, so the method is robust to prior choices.

## Data application

The method is validated by applying it to standard data examples. For all case studies, MCMC burn-in size is 6000, and no. of MCMC iterations is 10000. The training and test set size is of ratio 80/20. All data descriptions are in Table 3.

**Table 3:** Data Description

Data	Availability	N, P	Variables	Outcome
Pima Indians Diabetes	R package “mlbench” [40]	768,8	no. of times pregnant (*x*_1_), glucose (*x*2), pressure (*x*_3_), triceps: Triceps skin fold thickness (mm) (*x*_4_), insulin (*x*_5_), BMI (*x*_6_), pedigree (*x*_7_), Age (*x*_8_)	tested positive for diabetes (*y*=1), negative (*y*=0)
Colon	R package ‘HiDimDA’ [41]	62, 2000	human genes	40 tumor (*y*=1) & 22 normal (*y*=0) colon tissues
ADNI	Alzheimer’s Disease Neu-roimaging Initiative (ADNI) database (adni.loni.usc.edu) R package ADNIMERGE [42]	14712, 113, after pre-processing: 911, 22	Age, CDRSB_bl: Clinical Dementia Rating Sum of Boxes (core), ADAS11_bl: 11 item-AD Cognitive Scale (score), ADAS13_bl: 13 item-AD Cognitive Scale (score), MMSE_bl: Mini-Mental State Examination (score), RAVLT_immediate_bl, RAVLT_learning_bl, RAVLT_forgetting_bl, RAVLT_perc_forgetting_bl: Rey’s Auditory Verbal Learning Test (scores for immediate response, learning, forgetting and percentage forgetting), FAQ_bl: Functional Activities Questionnaire, APOE4: APOE4 gene presence, Hippocampus_bl: Volume of hippocampus, Ventricles_bl: Volume of ventricles, WholeBrain_bl: volume of Brain, Fusiform_bl: The volume of the fusiform gyrus, Entorhinal_bl: The volume of the entorhinal cortex, MidTemp_bl: The volume of the middle temporal gyrus, ICV: Intra Cranial Volume, PTGENDER: Participant’s gender, PTETHCAT: Participant’s ethnicity, PTRACCAT: Participant’s race, PTMARRY: Participant’s marital status	LR model: AD (*y*=1), EMCI or LMCI (*y*=0); MLR model: AD (*y*=2), EMCI (*y*=1), LMCI (*y*=0)
OASIS	Open Access Series of Imaging Studies (OASIS) [43]	373, 15; after pre-processing 373, 8	Visit: number of visits, gender, age, EDUC: Education, SES: Socioeconomic status as assessed by the Hollings head Index of Social Position, MMSE: Mini-Mental State Examination score, nWBV: Normalized whole brain volume, ASF: Atlas Scaling Factor	CDR: Clinical Dementia Rating (0 = no dementia, 0.5 = very mild AD, 1 = mild AD, 2 = moderate AD); For LR model: mild AD or AD (*y*=1), no dementia or very mild AD (*y*=0); For MLR model: mild AD or AD (*y*=2), no dementia (*y*=0), very mild AD (*y*=1)

### Pima indians diabetes

The data has been analyzed in the literature related to shrinkage priors, including [44–46]. The three priors can detect four variables by 95% credible intervals. The credible intervals of the coefficients with variable names “pregnant,” “glucose,” “mass (BMI),” and “pedigree (family history)” do not include zero; hence they are significant. These results are at par with [44]; the “pressure” variable is also determined as significant by DP prior. These three priors are also compared with Bayesian-Lasso(BLasso) [44], Bayesian-Elastic-Net(BElastic) [47] and frequentist methods such as Lasso, Elastic-Net and Ridge. Here, the three priors’ accuracy is similar, but BS is the least among all methods. Even though EN and Ridge have high accuracy, the frequentist methods have substantially high BS and low specificity values.

### Colon

This data comprises of gene expression levels for 40 tumors and 22 normal colon tissues measured for 6500 human genes using the Affymetrix oligonucleotide arrays [48]. Samples are obtained from tumor tissue and adjacent unaffected parts of the colon of the same patients [49]. Out of these, 2000 genes with the highest minimal intensity across the tissues were selected for classification purposes [48]. In the public domain, there is no demographic information available for the two groups such as race, age, sex distribution; else, a study could have been conducted about the benefits of inclusion of one or all these covariates in prediction. Lasso selects 12 genes. The priors are applied to the data with the lasso estimates as the initial values. Here, an approach of k-means similar to [18] with prostate cancer data set is adopted. The |*β*_*i*_| are clustered by K-means algorithm at each MCMC iteration into two clusters. For each iteration, the number of non-zero *β*’s is then estimated by the smaller cluster size out of the two clusters. A final estimate (F) of the number of non-zero signals is obtained by taking the highest frequency over all the MCMC iterations. The F-number of gene IDs are traced back for each iteration and the first F genes with the highest frequency are chosen. In the case of Horseshoe prior, it selects 36 genes with the highest frequencies, among which one gene (gene ID: 1423) is included among the top 20 genes selected by the t-test and fold change [50]. Also, the gene ID: 1325 selected by Lasso [51] can be seen in the set of 36 genes selected by Horseshoe. DL prior selects 164 genes, of which gene ID: 138 is included in the top 2 genes. DP selects 67 genes, out of which eight genes are in the set of genes selected by Horseshoe.The MCMC Gibbs sampling mixing and convergence were determined by trace plots and auto-correlation plots with an average adequate sample size of 23095.21 for Horseshoe, 39755.4 for DL, and 40129.35 for DP. About 95% of the genes conform to the Geweke diagnostic criteria for all three priors. A thinning step at every 15 steps of iteration did not significantly improve the results, so a posterior prediction analysis was performed on 40,000 iterations after burn-in. The MSE for HS, DL, and DP are 0.001,0.003,0.003, implying that MCMC has converged well. Here, on the basis of Brier score Horseshoe prior performs better than the other priors.

### ADNI

Alzheimer’s disease (AD) is a critical public health concern throughout the world and one of the most widespread neurodegenerative disorder [52]. AD is an irreparable brain disease, which impairs thinking and memory. Several machine learning methods have helped predict AD from Mild Cognitive Impairment (MCI); here, the shrinkage priors are used to predict AD from MCI, and they achieve high prediction measures.

This dataset is obtained from Alzheimer’s Disease Neuroimaging Initiative (ADNI) database (adni.loni.usc.edu). The ADNI was launched in 2003 as a public-private partnership, led by Principal Investigator Michael W. Weiner, MD. The main mission was to test if the combination of serial magnetic resonance imaging (MRI), positron emission tomography (PET), other biological markers, and clinical and neuropsychological assessment can be used to measure the progression of mild cognitive impairment (MCI) and early Alzheimer’s disease (AD).

The data used here is obtained from the R package ADNIMERGE [42] which is a subset of the “adnimerge” data that contains only baseline variable measurements (i.e., the first visit for each participant) that has a diagnosis. The “adnimerge” datas et merges several key variables from various case report forms and biomarker lab from all ADNI protocols (ADNI1, ADNIGO, and ADNI2). The integrated data consists of 113 variables and 14712 observations, which include multiple observations per participant, representing multiple visits to the study’s site for participant evaluation The participants is divided into five different classes, namely, Cognitively Normal (CN =4428), Early Mild Cognitive Impairment (EMCI =2687), Late Mild Cognitive Impairment (LMCI =4993), Subjective Memory Complaint (SMC =938), and Alzheimer’s Disease (AD =1654). After pre-processing, there were 911 observations, and 23 variables including the outcome variable. The demographic, cognitive assessment and clinical assessment attributes that are included in the analysis are described in [53]. The missing values are discarded, and the data is normalized. The three priors, along with Bayesian lasso and Bayesian EN, are used to predict AD from MCI that comprised of both early and late stages. The three priors achieve 85% accuracy and 93.5% specificity for the prediction of AD from the MCI stage and have comparable results with other shrinkage priors. The results also indicate that the most distinguishing attribute for the prediction of AD includes the ADAS13.bl (13 item AD Cognitive Scale score), RAVLT.perc.forgetting.bl (Rey’s Auditory Verbal Learning Test (scores for immediate response learning, forgetting and percentage forgetting)) by all three priors. FAQ.bl (Functional Activities Questionnaire) cognitive test was selected only by DP prior.

### OASIS

**Fig. 3:**
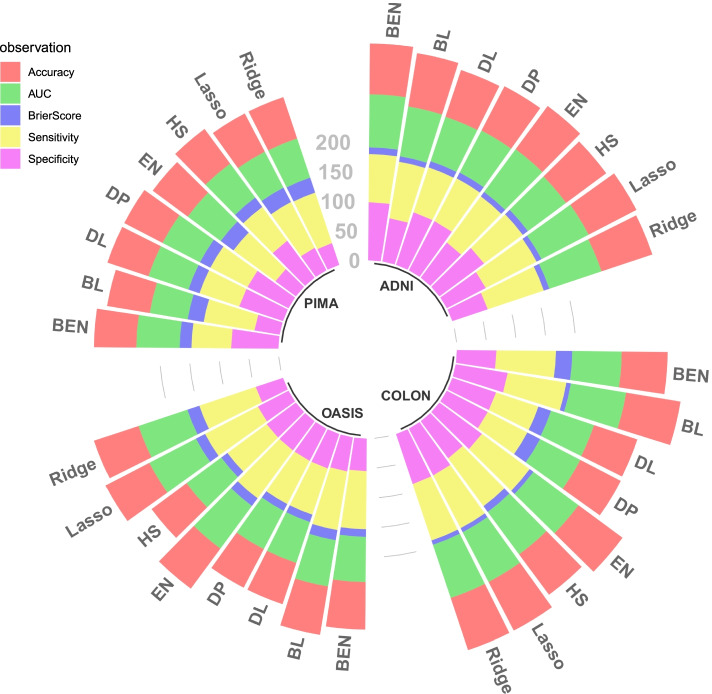
Circular Bar Chart comparing Prediction Metrics among data sets

**Fig. 4:**
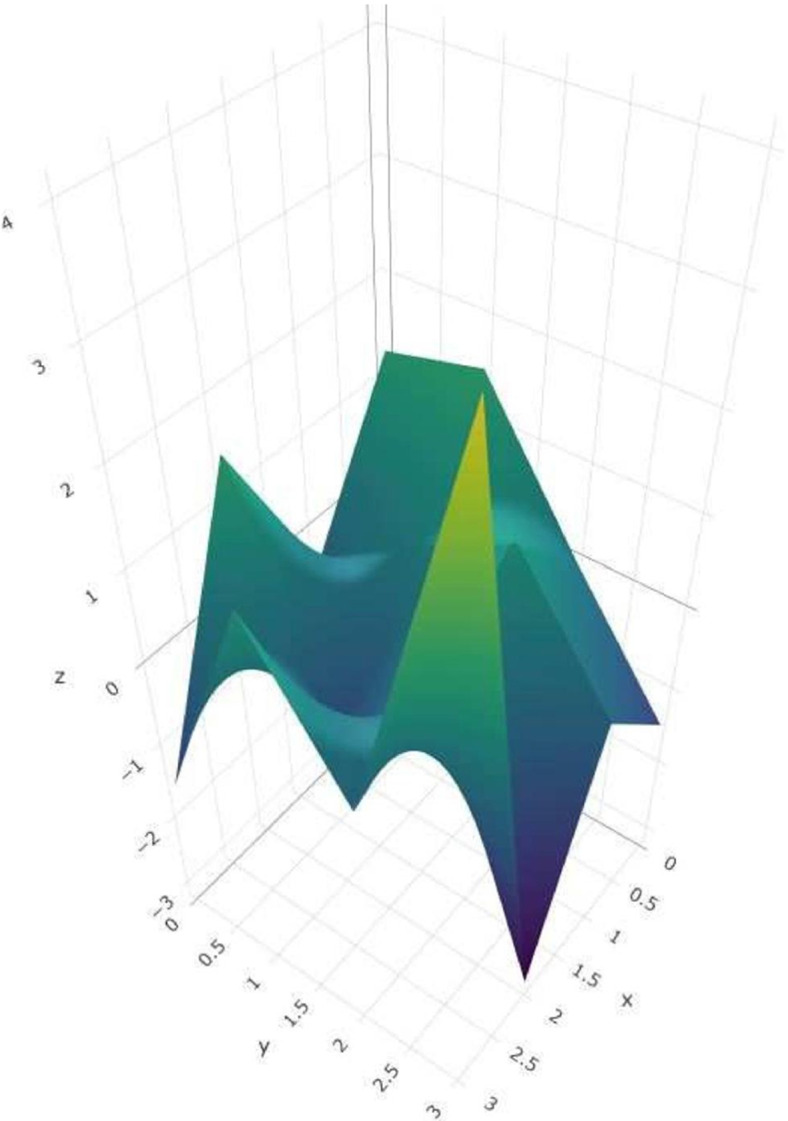
ROC Surface Plot for Shrinkage priors in ADNI data

This data set consists of a longitudinal collection of Non-demented and Demented Older Adults, 150 subjects aged 60 to 96. The three priors’ most frequently included variables are Age, SES, MMSE, gender, ASF, and nWBV. The three priors’ prediction efficiency shows that they outperform other methods concerning BS and specificity. Table 4, and the circular bar chart Fig. 3 gives a detailed view of the prediction performance of LR model. Figure 3 is efficient in describing the comparison of all the four data sets in a single platform. For each data set, eight bars correspond to the eight shrinkage methods, and each stacked bar consists of the five measures of prediction. For ADNI and PIMA, all methods perform similarly, though the priors do not prove to be efficient in the high-dimensional Colon data. For the MLR model, in ADNI data, the three classes are considered as EMCI, LMCI, and AD, and collected data for these classes had a dimension of 1438×113. A 10- fold cross-validation was used for prediction analysis. The variables selected by the priors are “CDRSB.bl,” “MMSE.bl,” “WholeBrain.bl”, “Fusiform.bl”, and “ICV,” which are real-life markers for AD prediction and are validated by previous literature. The five-category model did not have a high performance in terms of accuracy. DL and DP provide better metrics than Horseshoe here. For OASIS data, the categorical response variable is classified into three classes. The moderate (1) and mild CDR (2) ratings are combined since there were only four observations for the moderate class. The variables selected are Age, Education, Socio Economic Status (SES), Mini-Mental State Exam (MMSE), and normalized Whole Brain Volume (nWBV)”. Accuracy and AUC are presented in parentheses for the three priors in Table 4. Figure 4 is prepared by the R packages HUM [54], and plotly [55] that provides details about the behavior of the shrinkage priors. The ROC surface for horseshoe prior is presented here; all the other priors follow similarly.

**Table 4:** Prediction Performance in Real Life Data for LR

	Priors										
Measures	Horseshoe	DL	DP	BLasso	BElastic	Lasso	EN	Ridge	Gradient-Boosting	Random Forest	BART
	ADNI										
Accuracy	0.842 (0.736)	0.831 (0.747)	0.842 (0.747)	0.907	0.852	0.907	0.907	0.902	0.918	0.923	0.907
Sensitivity	0.810	0.796	0.810	0.964	0.810	0.964	0.964	0.993	0.971	0.985	0.964
Specificity	0.935	0.935	0.935	0.739	0.978	0.739	0.739	0.630	0.761	0.739	0.739
AUC	0.798 (0.880)	0.789 (0.884)	0.798 (0.883)	0.851	0.894	0.894	0.894	0.928	0.866	0.862	0.851
Brier Score	0.107	0.109	0.110	0.093	0.113	0.093	0.093	0.098	0.082	0.077	0.093
	OASIS										
Accuracy	0.733 (0.741)	0.733 (0.688)	0.733 (0.741)	0.827	0.800	0.827	0.840	0.787	0.760	0.760	0.773
Sensitivity	0.818	0.818	0.818	1.000	0.977	1.000	1.000	1.000	0.841	0.841	0.864
Specificity	0.613	0.613	0.613	0.581	0.548	0.581	0.613	0.484	0.645	0.645	0.645
AUC	0.727(0.764)	0.727 (0.701)	0.727(0.764)	0.790	0.763	0.886	0.893	0.867	0.756	0.756	0.772
Brier Score	0.129	0.130	0.129	0.173	0.126	0.173	0.160	0.213	0.240	0.240	0.227
	Pima Indian Diabetes										
Accuracy	0.727	0.727	0.727	0.727	0.708	0.727	0.734	0.734	0.786	0.792	0.786
Sensitivity	0.705	0.705	0.705	0.867	0.667	0.867	0.876	0.895	0.864	0.893	0.874
Specificity	0.776	0.776	0.776	0.429	0.796	0.429	0.429	0.388	0.627	0.588	0.608
AUC	0.711	0.711	0.711	0.648	0.731	0.682	0.692	0.696	0.746	0.741	0.741
Brier Score	0.197	0.197	0.197	0.273	0.199	0.273	0.266	0.266	0.214	0.208	0.214
	Colon										
Accuracy	0.846	0.769	0.769	0.923	0.769	0.923	0.923	0.923	0.846	0.846	0.692
Sensitivity	1.000	0.750	0.750	1.000	1.000	1.000	1.000	1.000	0.667	0.667	0.333
Specificity	0.778	0.778	0.778	0.889	0.667	0.889	0.889	0.889	0.900	0.900	0.900
AUC	0.889	0.764	0.764	0.944	0.833	0.944	0.944	0.944	0.783	0.783	0.567
Brier Score	0.121	0.224	0.240	0.077	0.276	0.077	0.077	0.077	0.154	0.154	0.308

The convergence metrics, trace plots, and acf plots for all data sets to assess the sampling procedure are presented in the supplementary file. The Geweke diagnostic (Z) is calculated that compares the mean of the samples drawn from the end of a chain of MCMC output to the mean of the samples at the beginning of the chain using a z-test statistic. A cut-off of 2 is arbitrarily chosen for the absolute value of Z. The percentage of |*Z*|<=2 is reported for each of the three priors.

Overall, it is seen that on two out of four datasets, our proposed method with the horseshoe prior outperformed the frequentist methods on specificity by as much as 10 percentage points. On the other two datasets, while we are behind on the specificity, we compare much favorably on sensitivity. The colon dataset has the second highest AUC from our method only superseded by Blasso. Overall, our method is clearly competitive and shows superiority in at least one of the measures over the frequentist methods in all datasets.

## Discussion

Bayesian shrinkage models can be utilized as a practical and useful alternative classification approach and a plausible way to select genetic markers and risk factors. This is a detailed study on a wide variety of settings comparing the three most well-known shrinkage priors. All of the three priors were able to recognize patterns differentiating the binary classes to a highly accurate level. This was shown by the excellent predictive performance of the binary LR model on the simulated data sets and fairly high predictive accuracies on a wide array of public health data sets. Some of these datasets were high dimensional, which exhibits the model’s power to scale up to these challenging scenarios. The algorithm is efficient, and the time taken to execute the simulations is relatively low, such as algorithm with *n*=500;*p*=50 takes about 673 seconds. This combination of computational power and predictive performance makes it a very reasonable method of use for practitioners who require quick high dimensional analysis, retaining the advantages of Bayesian analysis. In future we would formally explore the comparison with priors such as Spike and Slab priors and schemes ase the MH algorithm. We posit that features such as selections of indicators and acceptance-rejection step would not favorably compare with the proposed algorithm. We also extended the model to a multinomial logistic model that handles multiple categories. An R package “ShrinkageBayesGlm” is developed to be publicly available soon. We expect that the coming years will witness its wider dissemination among public health research and will be useful for predicting occurrences of common disorders as dementia, colon cancer, diabetes, etc. Computational advances, especially in high-dimensional cases [56] will continue expanding the scope of exact methods.

## Supplementary Information

**Additional file 1** The additional tables and figures are presented in the Supplementary file.

## Data Availability

The datasets used and/or analysed during the current study are available and information about it is included in this article in Table 3.

## Abbreviations

SSVS: Stochastic Search Variable Selection
GL: global-local
HS: Horseshoe
DL: Dirichlet Laplace
DP: Double Pareto
PG: Polya-Gamma
EN: Elastic Net
BE: Bayesian Elastic Net
BL: Bayesian Lasso
LR: Logistic Regression
MLR: Multinomial Logistic Regression
DA: Data Augmentation
MCMC: Markov Chain Monte Carlo
AUC: Area Under Curve
ROC: Receiver Operating Characteristic Curve
MSE: Mean Squared Error
BS: Brier Score
VS: variable selection
