# Supplementary material for: Applications of Bayesian shrinkage prior models in clinical research with categorical responses
Source: BMC Med Res Methodol. 2022 Apr 28;22:126. doi: 10.1186/s12874-022-01560-6 (PMC9046716; doi:10.1186/s12874-022-01560-6)
Supplement: Supplementary file 1 — Additional file 1 The additional tables and figures are presented in the Supplementary file. [file 12874_2022_1560_MOESM1_ESM.zip › Supplementary File.pdf]

Applications of Bayesian Shrinkage prior models in clinical research with categorical responses

Arinjita Bhattacharyya[1], Subhadip Pal[1†], Riten Mitra[1†], Shesh N. Rai[1,2,3,4,5*]

Supplementary Table S1

Geweke Diagnostics (%)

Dataset	Horseshoe	Dirichlet Laplace	Double Pareto
Pima Indian Diabetes	100	100	87.5
Colon	95.2	95.7	95.3
ADNI	90.9	95.5	86.3
OASIS	100	100	100

Supplementary Figure S1

Supplementary Figure S2

Supplementary Figure S3

Supplementary Figure S4
