# Supplementary figures and images for: Applications of Bayesian shrinkage prior models in clinical research with categorical responses
Source: BMC Med Res Methodol. 2022 Apr 28;22:126. doi: 10.1186/s12874-022-01560-6 (PMC9046716; doi:10.1186/s12874-022-01560-6)

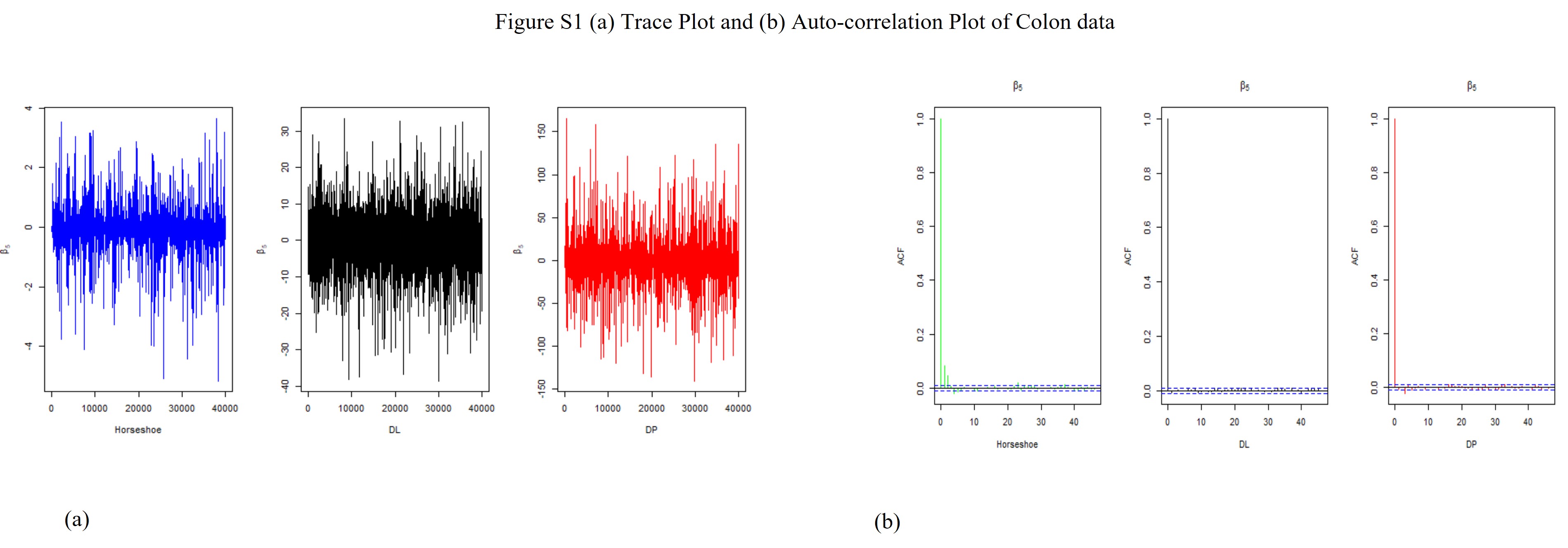

Supplement: Supplementary file 1 — Additional file 1 The additional tables and figures are presented in the Supplementary file. [file 12874_2022_1560_MOESM1_ESM.zip › Supplementary Figure S1.jpg]

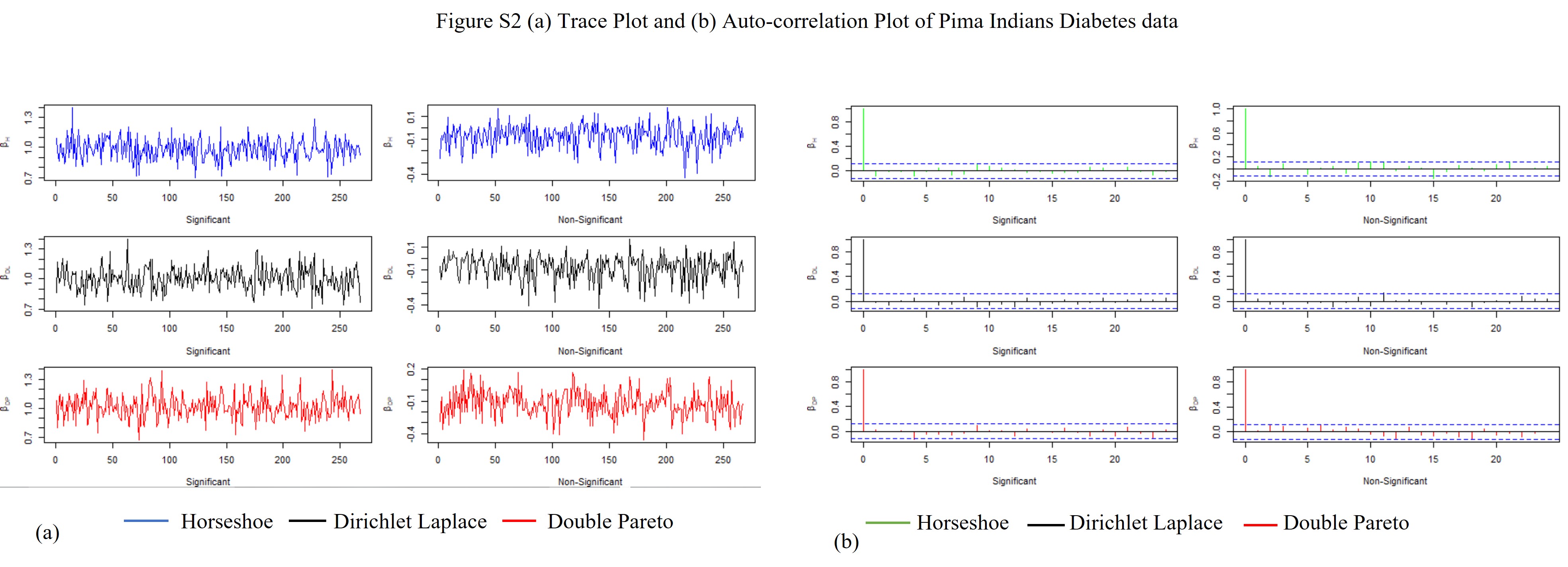

Supplement: Supplementary file 1 — Additional file 1 The additional tables and figures are presented in the Supplementary file. [file 12874_2022_1560_MOESM1_ESM.zip › Supplementary Figure S2.jpg]

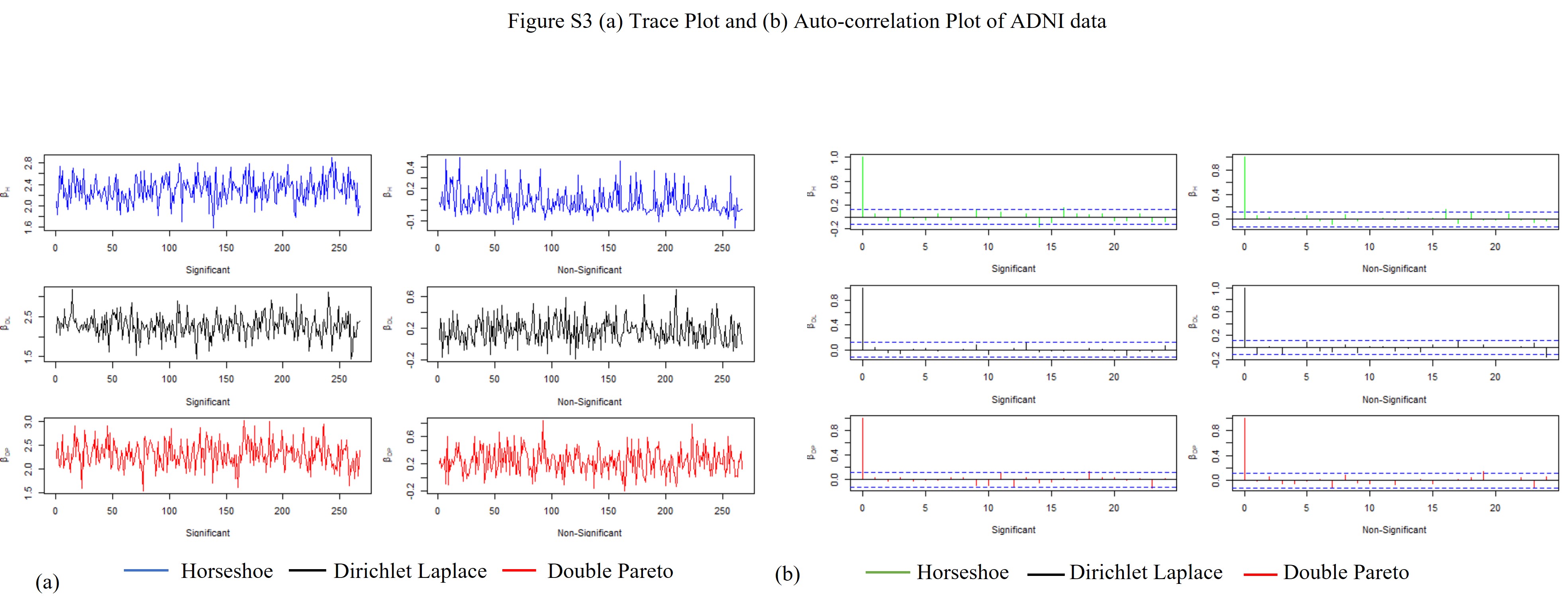

Supplement: Supplementary file 1 — Additional file 1 The additional tables and figures are presented in the Supplementary file. [file 12874_2022_1560_MOESM1_ESM.zip › Supplementary Figure S3.jpg]

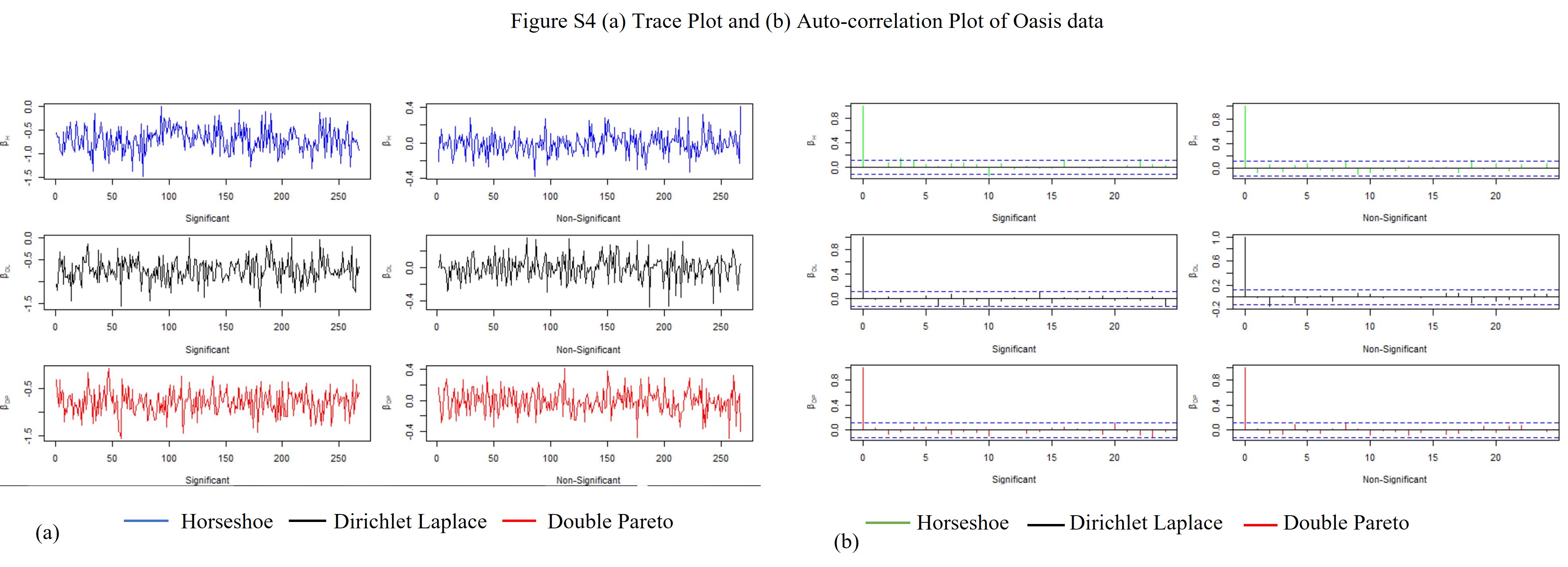

Supplement: Supplementary file 1 — Additional file 1 The additional tables and figures are presented in the Supplementary file. [file 12874_2022_1560_MOESM1_ESM.zip › Supplementary Figure S4.jpg]
